# Management of kratom-induced dyspigmentation with the 1064-nm picosecond laser

**DOI:** 10.1016/j.jdcr.2026.06.014

**Published:** 2026-06-13

**Authors:** Sanah Basrai, Gabrielle Brody, Mandy Majidian, Ray Jalian, Jeremy Man

**Affiliations:** a Kaiser Permanente Bernard J. Tyson School of Medicine, Pasadena, California; b Department of Dermatology, Kaiser Permanente Los Angeles Medical Center, Los Angeles, California

**Keywords:** dyspigmentation, kratom, picosecond laser

## Introduction

Kratom (*Mitragyna speciosa*), a psychoactive plant native to Southeast Asia, has been popularized in Western markets for the management of opioid withdrawal and chronic pain. This herbal supplement is widely accessible over the counter, allowing for unregulated use by consumers. Kratom has been associated with several unintended side effects, including hepatotoxicity, neurotoxicity, and cutaneous dyspigmentation, especially in sun-exposed areas.^1^, ^2^, ^3^ Although the mechanism remains unclear, confirmed cases of kratom-induced dyspigmentation remain rare and have limited response to topical treatment.^3^^,^^4^ Recently, Alhadyani et al^5^ described 2 cases of successful treatment with the 730-nm picosecond titanium-sapphire laser.

In this case report, to our knowledge, we present the first documented use of the 1064-nm picosecond laser for the treatment of kratom-induced dyspigmentation. The 1064-nm picosecond lasers are commonly used for the treatment of dermal pigment and tattoo ink because of their ability to target deeper melanin deposits while minimizing damage to the surrounding epidermis. Our treatment approach draws from the successful use of this modality in other drug-induced pigmentary disorders, such as minocycline-induced dyspigmentation.^6^ This report investigates the safety and efficacy of using a 1064-nm picosecond laser for kratom-related dyschromia, contributing to the growing but limited body of knowledge on the management of this rare but increasingly common condition.

## Case report

A 48-year-old man with a history of substance use disorder in the setting of chronic orthopedic pain presented with diffuse blue-to-gray dyspigmentation over sun-exposed areas, including his face, neck, and arms. He had been taking kratom daily since 2013 and first noticed dyspigmentation in 2017. He works outdoors, and he denied ingesting any oral silver solution.

**Fig 1:**
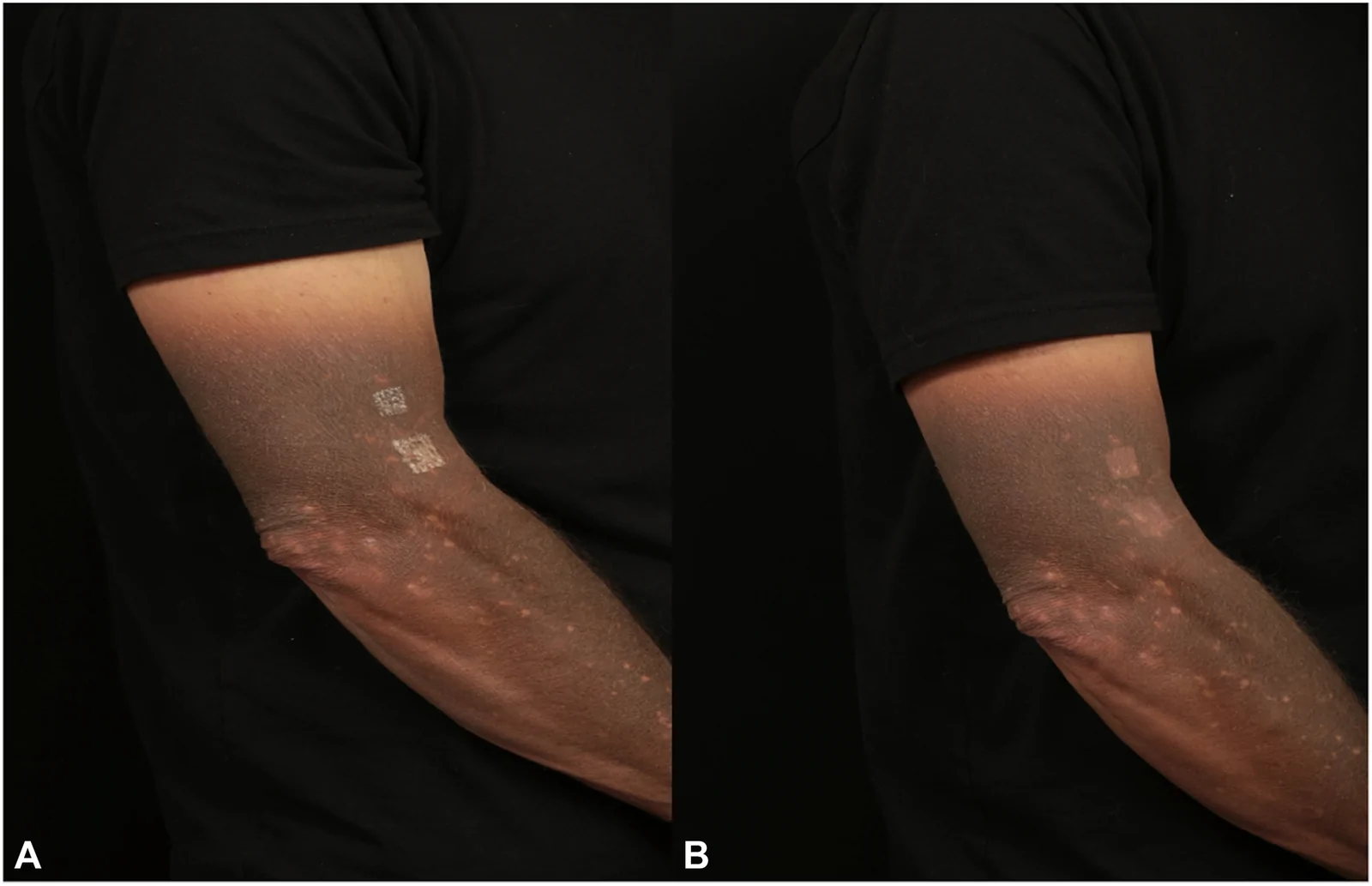
Immediately after (**A**) and 4 weeks after (**B**) treatment with the Cutera Enlighten III 1064-nm picosecond laser (Cutera, Inc.).

**Fig 2:**
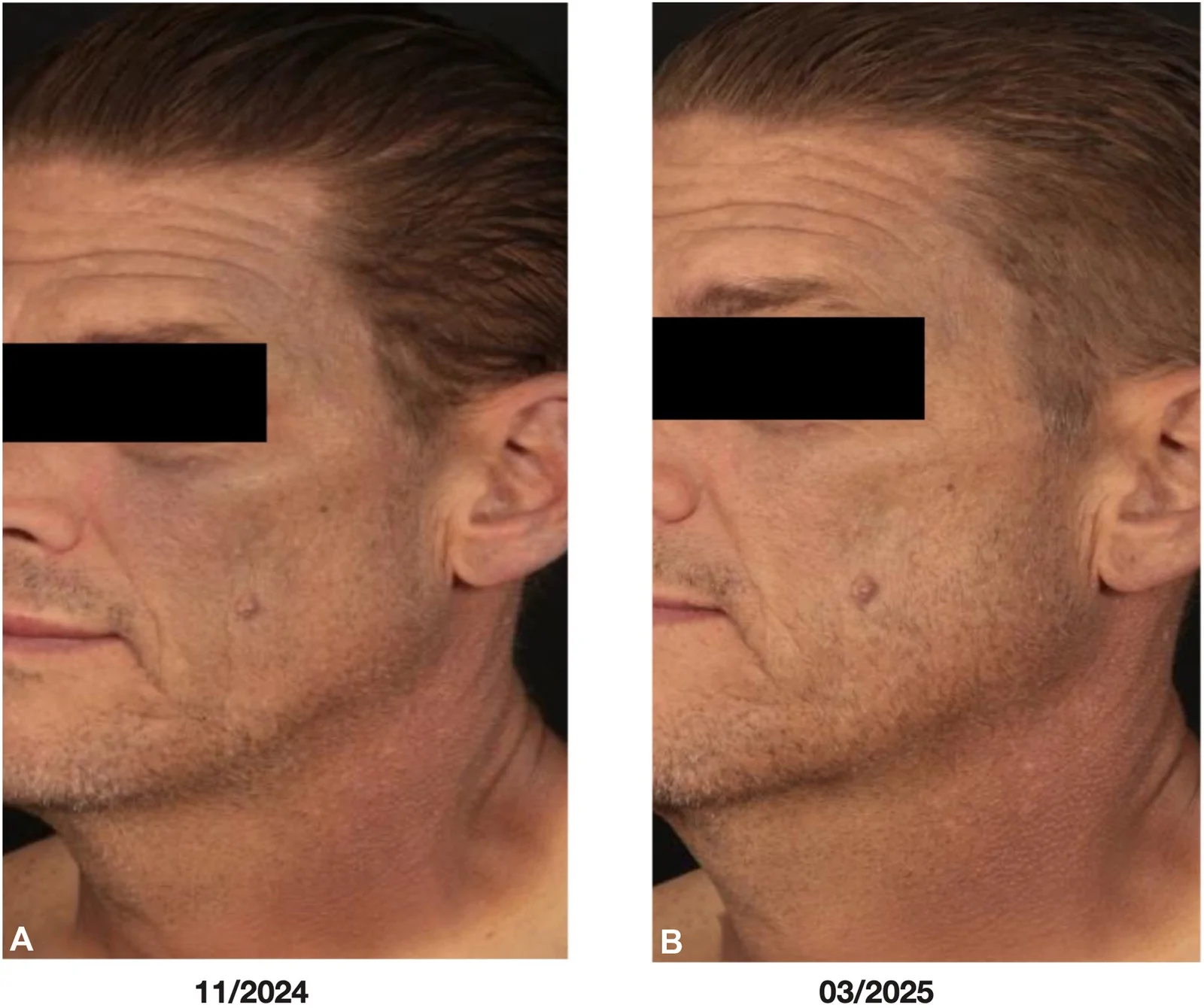
Before treatment of face (**A**) and 4 months after (**B**) treatment with the Cutera Enlighten III 1064-nm picosecond laser (Cutera, Inc.).

Test spots were performed using a picosecond laser at 670 nm and 1064 nm wavelengths. The patient reported mild-to-moderate pain throughout the procedure. Given a favorable clinical response and a lower risk of postinflammatory hyperpigmentation, treatment was initiated using the 1064 nm setting (Fig 1). A 1064-nm Nd:YAG picosecond laser was used with a spot size of 6 to 8 mm and fluences ranging from 1.0 to 2.4 J/cm^2^, titrated to achieve the clinical end point of immediate dermal whitening. Additionally, treatment was performed in sections (eg, hands 1 day, forearms another, etc). Significant lightening was achieved without postinflammatory hyperpigmentation; however, the patient was lost to follow-up (Fig 2).

## Conclusion

We present a case of kratom-associated dyspigmentation showing promising results with a 1064-nm laser. Although rare and often underrecognized, kratom-induced dyspigmentation is a documented consequence of prolonged kratom use.^2^^,^^3^ Its pathogenesis remains unclear, but the proposed mechanisms include melanogenesis secondary to μ-opioid receptor stimulation and antagonism of dopamine receptors.^3^^,^^7^

Topical therapies have largely been ineffective; for example, Johnson et al^4^ reported no clinical improvement after a 3-month course of hydroquinone 12%, kojic acid 6%, ascorbic acid 1%, and niacinamide 2%. In contrast, laser therapies show greater promise. Alhadyani et al have recently reported up to 98% pigment clearance in 1 patient using a 730-nm picosecond titanium-sapphire laser.

Our case further supports the therapeutic potential of laser-based interventions, specifically a 1064-nm wavelength picosecond laser, in treating kratom-related dyschromia. Although broader studies and extended follow-up are needed to validate these findings, our experience underscores laser therapy as a promising, targeted treatment approach for this uncommon pigmentary disorder.

## Conflicts of interest

None disclosed.
